# Light-driven oxidation of CH_4_ to C_1_ chemicals catalysed by an organometallic Ru complex with O_2_†

**DOI:** 10.1039/d2ra01772e

**Published:** 2022-04-28

**Authors:** Tatsuya Nakano, Tsukasa Abe, Takahiro Matsumoto, Kento Kimura, Genta Nakamura, Shinya Hayami, Yoshihito Shiota, Kazunari Yoshizawa, Seiji Ogo

**Affiliations:** Department of Chemistry and Biochemistry, Graduate School of Engineering, Kyushu University 744 Moto-oka, Nishi-ku Fukuoka 819-0395 Japan matsumoto.takahiro.236@m.kyushu-u.ac.jp; Institute for Materials Chemistry and Engineering, Kyushu University 744 Moto-oka, Nishi-ku Fukuoka 819-0395 Japan; International Institute for Carbon-Neutral Energy Research (WPI-I2CNER), Kyushu University 744 Moto-oka, Nishi-ku Fukuoka 819-0395 Japan; Precursory Research for Embryonic Science and Technology (PRESTO), Japan Science and Technology Agency (JST) Kawaguchi 332-0012 Japan; Graduate School of Science and Technology, Kumamoto University 2-39-1 Kurokami, Chuo-ku Kumamoto 860-8555 Japan

## Abstract

CH_4_ conversion is one of the most challenging chemical reactions due to its inertness in terms of physical and chemical properties. We have achieved photo-induced C–H bond breaking of CH_4_ and successive C–O bond formation to form CH_3_OH concomitant with HCHO by an organometallic Ru complex with O_2_.

## Abstract 2

We have achieved aerobic transformation of methane to C_1_ chemicals catalysed by a homogeneous organometallic catalyst with light energy input.

## Graphical abstract

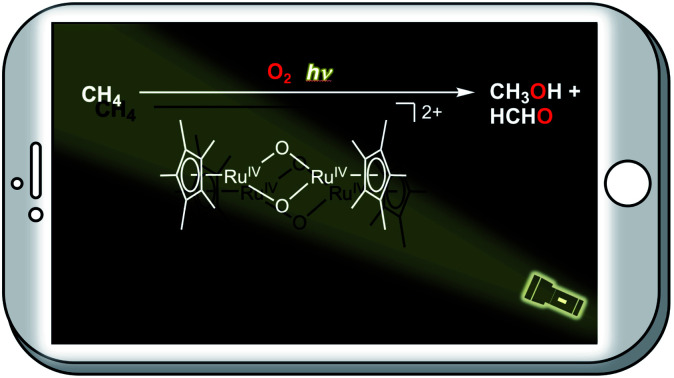

CH_4_ is one of the most promising resources of energy and materials because it has high affinity with renewable energy, and has become capable of being easily and abundantly obtained in biomethane form from biomass by means of recent technological developments.^1,2^ In order to use CH_4_ in industrial processes instead of naphtha, innovative and useful transformation methods are now strongly demanded. However, its inertness in view of its physical and chemical properties makes CH_4_ one of the most unreactive molecules.^3^ To date, there have been three type of catalysts, *i.e.*, enzymatic, heterogeneous and homogeneous, found for the direct oxidation of CH_4_ to CH_3_OH with O_2_ as an oxidant.^4–7^ Soluble and particulate methane monooxygenases (sMMOs and pMMOs) are well-known enzymes that oxidise CH_4_ to CH_3_OH with O_2_ under ambient conditions. Their active sites are constructed from Fe and Cu centres for sMMOs and pMMOs, respectively.^4^ They cleave the unreactive C–H bond of CH_4_ with subsequent C–O bond formation, proposed to be promoted by the Fe and Cu oxido species. By mimicking the active-site structure of sMMOs and pMMOs, heterogeneous catalysts^5,6^ and homogeneous Cu catalysts^7^ have been developed to catalyse aerobic CH_4_ oxidation to CH_3_OH. The heterogeneous zeolite catalysts need high temperature, and the homogeneous Cu catalysts need H_2_O_2_ as a reductant for the catalytic reaction. These catalysts may possess metal oxido cores that can promote C–H bond activation like MMOs. In addition to the MMO-inspired catalysts, a homogeneous inorganic compound of ClO_2_ works as a light-triggered oxidizing reagent to convert CH_4_ to CH_3_OH and HCOOH with O_2_ in a non-catalytic system.^8^ Homogeneous organometallic complexes other than CH_4_-to-CH_3_OH catalysts have also been designed for the conversion of CH_4_ to various significant compounds, capitalizing on the flexible and designable tuning of the ligand environment surrounding the metal centre(s).^9^ Recently, various heterogeneous catalysts have also been developed for CH_4_ conversion.^6^ While many efforts have been made to date for catalytic CH_4_ conversion, the direct catalytic conversion of CH_4_ to C_1_ chemicals of CH_3_OH and HCHO by a homogeneous organometallic catalyst with light irradiation has not yet been reported. Here, we report aerobic CH_4_ oxidation to CH_3_OH and HCHO catalysed by a homogeneous Ru complex in water with input of light energy. Most photocatalysts of organometallic complexes have been developed for redox reactions that mean single electron transfer between metal complexes and external electron donors/acceptors.^10^ Recently, charge transfers, such as ligand-to-metal or metal-to-ligand originating from organometallic complexes, have been utilized for chemical reactions such as material transformations apart from single-electron transfer reactions.^9*g*,11^ This advanced method should expand the possibility of photo-induced organometallic catalysis. We have developed a novel photo-driven C–H activating catalyst by means of charge transfer derived from a homogenous Ru complex.

A water-soluble and oxygen-sensitive Ru^II^ complex, [Ru^II^(η^5^-C_5_Me_5_)(H_2_O)_3_]^+^ (1), was oxygenated by O_2_ in H_2_O to rapidly generate oxidised species like a bis(μ-oxido) Ru_2_^IV^ species, [Ru_2_^IV^(η^5^-C_5_Me_5_)_2_(μ-O)_2_]^2+^ (2) (Fig. 1). This species was not formed in CH_3_CN but formed in H_2_O, which was likely to be caused by stabilization of the Ru_2_^IV^(μ-O)_2_ core in a polar environment. Density functional theory (DFT) calculations indicated that 2 was stabilized in H_2_O but destabilized under vacuum conditions relative to the corresponding starting Ru^II^ triaqua complex 1 (Fig. S1†). The oxygenated species 2 is stable in H_2_O at ambient temperature unlike bis(μ-oxido) Fe_2_ species that are generally unstable at ambient temperature.^12^ Its stability gave us a chance to irradiate 2 with light to form a highly active excited state.

**Fig. 1:**
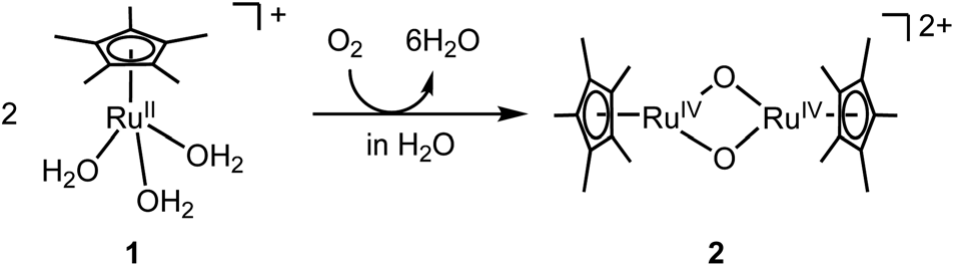
Synthesis of bis(μ-oxido) Ru_2_^IV^ complex 2 from oxygenation of mononuclear Ru^II^ triaqua complex 1 in H_2_O.

The structure of 2 was estimated by electrospray ionization-mass spectrometry (ESI-MS) (Fig. S2 and S3†) and DFT calculations (Fig. 2). The positive-ion ESI mass spectrum of 2 in H_2_O shows a prominent signal at *m*/*z* 521.9 that corresponds to [2 + OH]^+^, and a characteristic isotopic distribution that matches well with the calculated isotopic distribution (Fig. S2a–c†). It can be strongly suggested that complex 2 bears oxido ligands by isotope-labelling experiments using O_2_ in H_2_^18^O and ^18^O_2_ in H_2_O during oxygenation of 1. The positive-ion ESI mass spectrum obtained from the reaction of 1 with O_2_ in H_2_^18^O shows a prominent signal at *m*/*z* 527.9 that corresponds to [^18^O-labeled 2 + ^18^OH]^+^ (Fig. S2d†), while the positive-ion ESI mass spectrum obtained from the reaction of 1 with ^18^O_2_ in H_2_O shows a prominent signal at *m*/*z* 521.9 that corresponds to [2 + OH]^+^ (Fig. S2e†). These labelling experiments clearly reveal the presence of water-exchangeable ligands in 2 (Fig. S3†), which means that the oxido ligands should be coordinated to the Ru^IV^ centre. It is well known that oxido ligand(s) coordinating to metal centre(s) can be easily exchanged by external water.^13^ The high-valent metal centre is likely to bind an oxido ligand rather than a hydroxido ligand because such an oxido ligand has little ability to accept a proton to form a hydroxido ligand, caused by the Lewis basicity of the oxido ligand necessarily being lowered by delocalizing the electron density of the electron-rich oxido ligand toward the electron-deficient high-valent metal centre.^12,14^

**Fig. 2:**
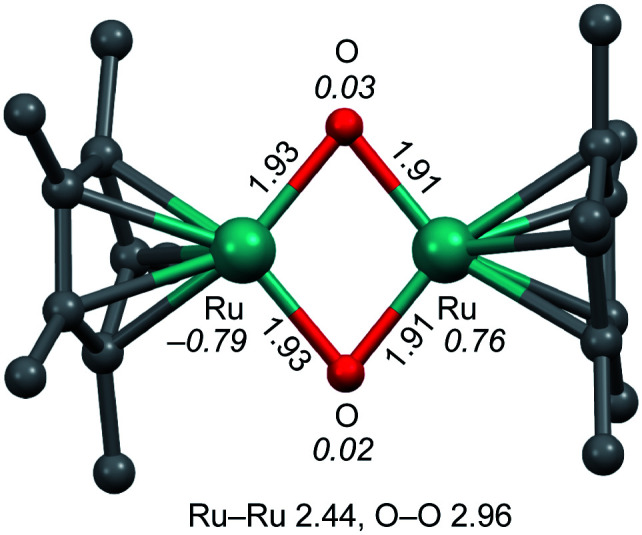
Optimized structure of bis(μ-oxido) Ru_2_^IV^ complex 2. The structure of 2 in the ground singlet state was optimized by DFT calculations. Units are in Å. The italicized values represent the spin densities of the Ru and O atoms. H atoms are omitted for clarity.

DFT calculations indicated that the optimized structure of 2 contains a bis(μ-oxido) Ru_2_^IV^ core rather than a (μ-peroxido) Ru_2_^III^ core, as shown in Fig. 2. Multinuclear Ru (hydr)oxido complexes have been reported, which are structurally similar to the Ru_2_(μ-O)_2_ centre of 2.^15^ The electron-donating η^5^-C_5_Me_5_ ligand allows the dinuclear Ru centre to access high-valent oxidation states of IV and the steric hindrance of the methyl groups of the η^5^-C_5_Me_5_ ligand creates a small cavity around the Ru atoms for the arrangement of only two oxido ligands. The Ru_2_^IV^(μ-O)_2_ structure seems to be characteristic of this ligand environment system. Changing ligand environments with respect to electronic effects and steric hindrance can provide various Ru^*x*^_2_{μ-O(H_*y*_)}_*z*_ structures (*x* = III–VI, *y* = 0–1, and *z* = 1–3).^15^ The distances of the two Ru centres and the two O atoms in 2 were calculated to be 2.44 and 2.96 Å (Fig. 2), respectively, which correspond to the interacting dinuclear Ru centres and the cleavage of the O–O bond. Spin density analysis indicates each Ru^IV^ centre has *S* = 1 and an interatomic interaction of two Ru^IV^ centres with the antiferromagnetic exchange interaction results in *S* = 0 in the ground state of 2 (^1^R, Table S1†), which is consistent with the experimental observation with a superconducting quantum interference device (SQUID) that the bis(μ-oxido) Ru_2_^IV^ complex 2 is diamagnetic. On the basis of experimental and DFT results, the bis(μ-oxido) Ru_2_^IV^ species 2 can be generated from four-electron reduction of O_2_ by two Ru^II^ centres *via* O–O bond breaking.

An ultraviolet-visible (UV-vis) spectral change from 1 to 2 by oxygenation in H_2_O shows a decrease in absorption bands around 330 nm (*ε* = 900 M^−1^ cm^−1^) and 400 nm (*ε* = 1300 M^−1^ cm^−1^) derived from 1 and an increase in a broad band around 290 nm (*ε* = 3800 M^−1^ cm^−1^) derived from 2 (Fig. S4†). Since the characteristic absorption band of 2 is observed in the UV region, we irradiated 2 with UV light for excitation. Time-dependent (TD)-DFT calculations are consistent with the experimental UV-vis spectra of 1 and 2 (Fig. S5†). The TD-DFT calculations of 2 show an absorption band at 263 nm, assigned to the charge transfer from the ground singlet state to the excited triplet state (Fig. S5b†). While the oxido ligands in 2 show little radical character with a spin density of almost zero (^1^R, Table S1†), the oxido ligands in the excited triplet state are capable of showing a radical character (^3^R*, Table S1†), described below in detail. This radical character must originate in the abstraction of an H atom from CH_4_ in the initiation step.

Following spectroscopic, mass-spectrometric and DFT analyses of 2, we investigated its photo-induced oxidation of CH_4_ in H_2_O. An aqueous solution of 2 under a CH_4_/O_2_ atmosphere (partial pressures of CH_4_ and O_2_ = 4 and 2 MPa, respectively) was irradiated by UV light (250–385 nm, 15 mW cm^−2^) for 5 h. Subsequently, the resulting aqueous solution was analysed by gas chromatography-mass spectrometry (GC-MS) after removing Ru complex(es) by passage through a silica gel column. CH_3_OH and HCHO were observed by GC-MS analysis (Fig. S6†), with their retention times and fragment patterns clearly corresponding to those of authentic CH_3_OH and HCHO. No HCOOH was observed by GC-MS. Control experiments were conducted without 2, UV light, CH_4_, or O_2_, all showing no product formation. When visible light (385–740 nm) was used instead of UV light, no reaction occurred. We determined the TONs of CH_3_OH and HCHO as 1.1 and 3.0, respectively; thus, the total TON was estimated to be 4.1. Considering that CH_3_OH was formed by 2-electron oxidation of CH_4_ with 2-electron oxidant 2 and CH_3_OH was 2-electron oxidized to form HCHO by 2, we calculated the TONs as follows: (mol of CH_3_OH)/(mol of 2) for CH_3_OH and (mol of HCHO) × 2/(mol of 2) for HCHO. Although the order of these TON values is the same as those of trinuclear Cu oxide systems that catalysed CH_4_ oxidation to CH_3_OH by O_2_ using H_2_O_2_ as reductant (TON = 1.4 or ∼6),^7^ our system needs only O_2_. We also determined the yields of CH_3_OH and HCHO based on CH_4_ to be 0.12 and 0.17%, respectively. We confirmed that photo-induced CH_3_OH oxidation yielded HCHO with 2 under the same conditions as the photo-induced CH_4_ oxidation. No HCOOH was also detected in the CH_3_OH oxidation. In order to confirm the origin of the oxygen atom of CH_3_OH, we conducted an isotope labelling experiment of photo-induced oxidation of CH_4_ by 2 with ^16^O_2_ in H_2_^18^O. No ^18^O-incorporated methanol (CH_3_^18^OH) was formed, but CH_3_^16^OH was observed. This result indicates that in the process of C–H bond activation of CH_4_, coupling of a CH_3_ radical with O_2_ occurs prior to OH rebound to the CH_3_ radical. After the C–H bond cleavage of CH_4_, DFT calculations indicate that the interaction of the CH_3_ radical with the OH ligand coordinating to the Ru centre is energetically higher than a transition state corresponding to the release of a CH_3_ radical from the (μ-hydroxido)(μ-oxido) Ru_2_^III,IV^ core (Fig. S7†). The insights, benefitting from the reports of activation of weaker C–H bonds in hydrocarbons rather than CH_4_ by metal oxido species without light irradiation, also permit us to propose H atom abstraction from the C–H bond of CH_4_.^12,14,16^

We followed the reaction of bis(μ-oxido) Ru_2_^IV^ species 2 with CH_4_ and O_2_ under light irradiation by ESI-MS (Fig. S8†). The ESI-MS results indicate that the main signal derived from 2 decreased as a signal at *m*/*z* 371.1, assignable to a tetramethylfulvene-coordinating Ru^II^ complex [Ru^II^(tetramethylfulvene)(η^5^-C_5_Me_5_)]^+^, and unidentified signals increased. The formation of tetramethylfulvene complex indicates that the methyl group of η^5^-C_5_Me_5_ was oxidized.^17^

Photo-driven oxidation of C_2_H_6_ by using 2 with O_2_ (partial pressures of C_2_H_6_ and O_2_ = 2 and 1 MPa, respectively) also occurs as in the case with CH_4_ oxidation. The products of C_2_H_5_OH and CH_3_CHO were observed by GC-MS (Fig. S9†), where their TONs were determined to be 0.31 and 0.46, respectively, based on the same calculation protocol as for CH_4_ oxidation. The total TON was calculated as 0.77. A trace amount of CH_3_COOH was observed in the C_2_H_6_ oxidation. The TON of C_2_H_6_ oxidation is slightly lower than that of the CH_4_ oxidation, which suggests that the oxidation reaction with 2 is relevant to the molecular size of the external substrate. The ten methyl groups of two η^5^-C_5_Me_5_ ligands seem to protect the bis(μ-oxido) Ru_2_^IV^ core, which allows a smaller molecule to access the active bis(μ-oxido) centre.

DFT calculations indicate that photo-excitation of 2 is required to cause H atom abstraction from CH_4_ (Fig. 3), which is consistent with the experimental result that 2 shows no reactivity toward CH_4_ without light irradiation. Fig. 3 shows the computed energy surfaces for the C–H bond activation by 2 in the open-shell singlet and triplet states. To obtain reaction coordinates of the C–H bond dissociation, we performed intrinsic reaction coordinate (IRC) calculations in the ground state. The potential energy surfaces of the excited states were obtained by a single-point calculation using the TD-DFT method along the reaction coordinate. The reactions involve the interaction of the oxido ligand with the H atom, followed by H atom abstraction from CH_4_ to generate a CH_3_ radical with the (μ-hydroxido)(μ-oxido) Ru_2_^III,IV^ species. Calculated activation energies for the C–H bond cleavage of CH_4_ by the catalyst are 33.4 kcal mol^−1^ in the ground state S_0_ and 19.6 kcal mol^−1^ in the triplet excited state T_27_. These results lead us to conclude that CH_4_ activation with 2 is likely to occur in the transition state in the potential energy surface of the excited state.

**Fig. 3:**
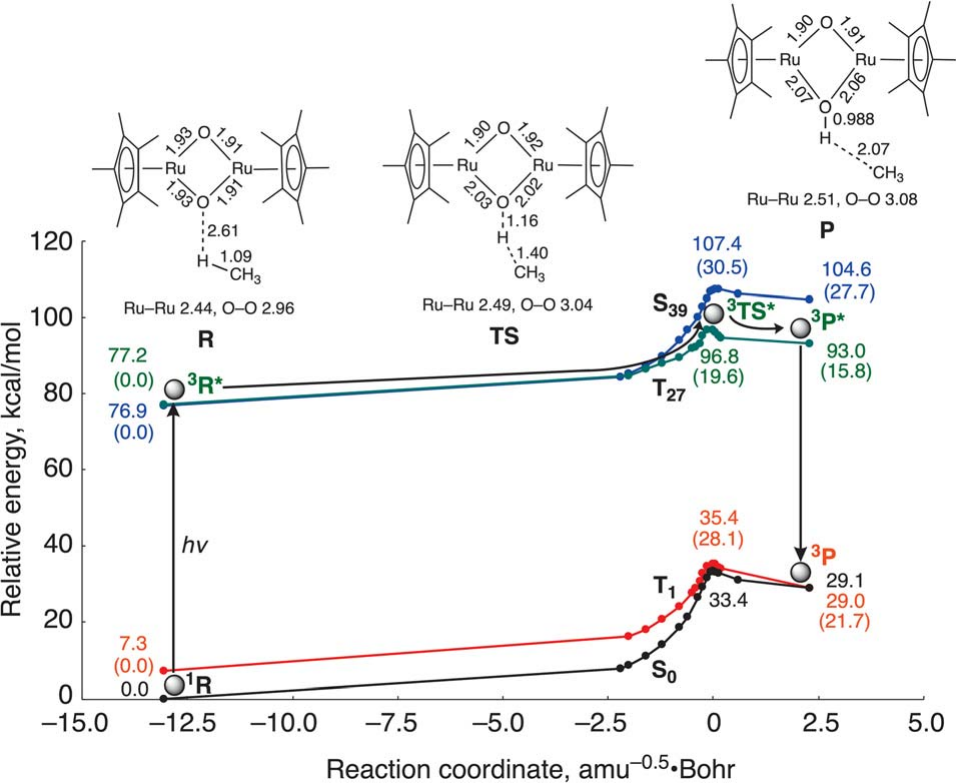
Computed energy surfaces for the C–H bond activation of CH_4_ by 2 in the ground state S_0_ and the three excited states T_1_, T_27_ and S_39_. R: reactant complex, TS: transition state and P: product complex. The values in parentheses are relative energies from R in each state. Distances and energies are given in units of Å and kcal mol^−1^, respectively.

Spin density analysis shows the oxido ligands of the reactant complex in the ground singlet state ^1^R have little radical character (O_1_: 0.01; O_2_: 0.03), while those in the excited triplet state ^3^R* have a more radical character (O_1_: 0.23; O_2_: 0.24) (Table S1†). The increase in the spin densities of the μ-oxido ligands in the bis(μ-oxido)dicopper complexes enhances the reactivity for H atom abstraction from CH_4_.^18^ Therefore, it is considered that the increase in the spin densities in the μ-oxido moieties diminishes the activation energy of H atom abstraction from CH_4_. We considered this the reason why the spin densities in the μ-oxido moieties increase by irradiation with UV light. Since the two unpaired electrons in the Ru centres are antiferromagnetically coupled in the ground singlet state of 2, the delocalized electrons of the μ-oxido moieties are cancelled. In contrast, UV light irradiation induces metal-to-metal charge transfer (MMCT) (Fig. S5†) to cause the spin inversion of an unpaired electron in the Ru centre. Therefore, the delocalized electrons of the μ-oxido moieties are enhanced, resulting in the radical character of the μ-oxido moieties. In the transition state (TS), the C atom of the CH_3_ radical increases in radical character with H atom migration, while the spin density of the Ru centre decreases. Thus, O–H bond formation and CH_3_ radical formation occur simultaneously.

On the basis of experimental analyses and DFT calculations, we propose a reaction mechanism of photo-induced CH_4_ oxidation by the Ru complex with O_2_ (Fig. 4). The bis(μ-oxido) Ru_2_^IV^ species 2 is excited by UV light to generate the excited species 3. The highly active excited species 3 is able to abstract an H atom from CH_4_ to afford (μ-hydroxido)(μ-oxido) Ru_2_^III,IV^ species 4 with the CH_3_ radical. The CH_3_ radical reacts with O_2_ to form a CH_3_OO radical, which can be coupled intermolecularly to generate a CH_3_OOOOCH_3_ species. This releases O_2_ to form a CH_3_O radical,^19^ which abstracts an H atom from 4 to afford CH_3_OH together with regeneration of 2. Based on thermodynamic energy calculations for CH_4_ oxidation to CH_3_OH with 2 in the ground state in H_2_O at standard temperature (eqn (1)–(4), the energies are corrected by zero-point vibrational energies and Gibbs free energies at 298.15 K), the process of H atom abstraction from CH_4_ to a CH_3_ radical is an endergonic reaction (Δ*G* = 28.0 kcal mol^−1^), although the processes of CH_3_ radical with O_2_ (Δ*G* = −27.0 kcal mol^−1^) and CH_3_O radical with 2 (Δ*G* = −24.9 kcal mol^−1^) are exergonic reactions. The overall reaction of CH_4_ with O_2_ to CH_3_OH is exergonic (Δ*G* = −23.9 kcal mol^−1^). Because only the first step of H atom abstraction by 2 needs external energy, we must input light energy into this system.

1 CH_4_ + 2 = CH_3_˙ + 4 − 28.0 kcal

2 CH_3_˙ + ½ O_2_ = CH_3_O˙ + 27.0 kcal

3 CH_3_O˙ + 4 = CH_3_OH + 2 + 24.9 kcal

(1) + (2) + (3):

4 CH_4_ + ½ O_2_ = CH_3_OH + 23.9 kcal

**Fig. 4:**
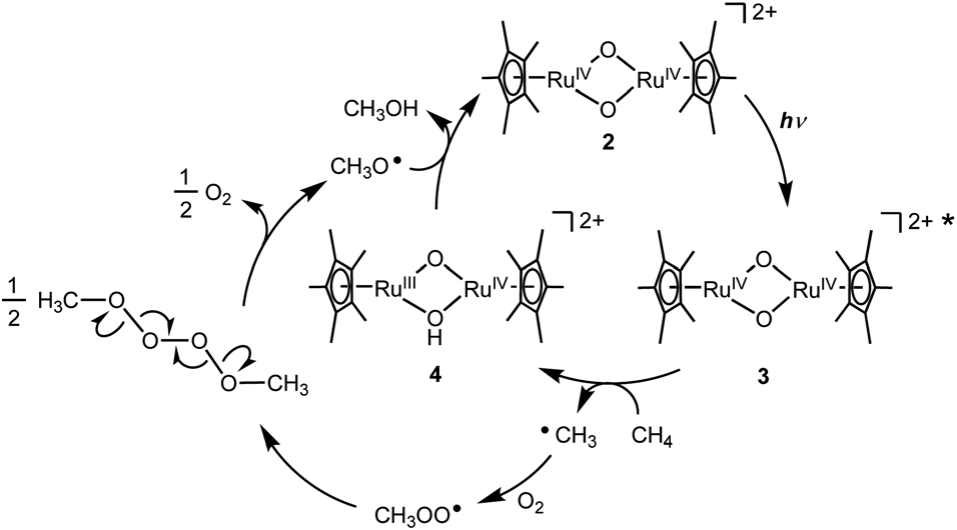
A proposed mechanism for the photo-induced oxidation of CH_4_ to CH_3_OH by Ru complex with O_2_ in H_2_O.

In conclusion, we have succeeded in the photo-induced conversion of CH_4_ to C_1_ chemicals of CH_3_OH and HCHO catalysed by the water-soluble bis(μ-oxido) Ru_2_^IV^ complex with O_2_. This is the first case of catalytic oxidation of CH_4_ to CH_3_OH and HCHO with a homogeneous catalyst by using only O_2_. The light-triggered radical character of the oxido ligands enables the activation of the unreactive C–H bond of CH_4_, as evidenced by experimental results and DFT calculations. We think it will be possible to apply such a photo-excited metal complex to the activation of various unactivated molecules.

## Conflicts of interest

There are no conflicts to declare.

## Supplementary Material

RA-012-D2RA01772E-s001
