# Supplementary material for: Light-driven oxidation of CH4 to C1 chemicals catalysed by an organometallic Ru complex with O2
Source: RSC Adv. 2022 Apr 28;12(20):12253–7. doi: 10.1039/d2ra01772e (PMC9050190; doi:10.1039/d2ra01772e)
Supplement: RA-012-D2RA01772E-s001 [file RA-012-D2RA01772E-s001.pdf]

Electronic Supplementary Material (ESI) for RSC Advances.
This journal is © The Royal Society of Chemistry 2022

Electronic Supplementary Information for

Light-Driven Oxidation of CH_4 to C_1 Chemicals Catalysed by an Organometallic Ru Complex with O_2

Tatsuya Nakano, Tsukasa Abe, Takahiro Matsumoto, Kento Kimura, Genta Nakamura,*

Shinya Hayami, Yoshihito Shiota, Kazunari Yoshizawa* and Seiji Ogo**

*Corresponding author. E-mail: matsumoto.takahiro.236@m.kyushu-u.ac.jp

Table of Contents

Experimental Section	page S1
Table S1	page S5
Fig. S1	page S6
Fig. S2	page S7
Fig. S3	page S8
Fig. S4	page S9
Fig. S5	page S10
Fig. S6	page S11
Fig. S7	page S12
Fig. S8	page S13
Fig. S9	page S14
Fig. S10	page S15
References	page S16

Experimental Section

Materials and Methods. All experiments were carried out under a N_2 atmosphere by using standard Schlenk techniques and a glovebox. CH_4 (99.9999%) was purchased from OSAKA GAS LIQUID Co., Ltd. C_2H_6 (>99.9%) and O_2 gases (99.9%) were purchased from Sumitomo Seika Chemical Co., Ltd. D_2O (99.9%) was purchased from Sigma-Aldrich. $^{18}O_2$ (98 atm%) was purchased from SI Science Co., Ltd. $H_2{}^{18}O$ (98 atm%) was purchased from Taiyo Nippon Sanso Co. H_2O was purchased from Hayashi Pure Chemical Ind., Ltd. A ruthenium complex $[Ru^{II}(\eta^5\text{-}C_5Me_5)(CH_3CN)_3](NO_3)$ was prepared by the methods described in the literature.[1]

^1H NMR spectra were recorded on a JEOL JNM-AL400 spectrometer. ^1H NMR experiments in D_2O were measured at 25 °C using 3-(trimethylsilyl)-propionic-2,2,3,3-d_4 acid sodium salt (TSP) as an internal standard. ESI-MS data were obtained on a JEOL JMS-T100LC AccuTOF. UV-vis spectra were recorded on an Agilent Technologies Cary 8454 at room temperature (light path length: 0.10 cm). GC-MS data were recorded on a SHIMADZU GCMS-QP 2010. The magnetic susceptibilities were measured with a SQUID magnetometer (Quantum Design MPMS-5S). UV and visible light irradiation were carried out using Asahi Spectra MAX 303. Light power was measured by a Newport 843-R power meter. High pressure device with sapphire window transmitting UV light was made by Syn Corporation Ltd.

$[Ru^{II}(\eta^5\text{-}C_5Me_5)(H_2O)_3](NO_3)$ {[1](NO₃)}. $[Ru^{II}(\eta^5\text{-}C_5Me_5)(CH_3CN)_3](NO_3)$ was dissolved in H_2O, resulting in replacement of the CH_3CN ligands with H_2O to form a Ru aqua complex, $[Ru^{II}(\eta^5\text{-}C_5Me_5)(H_2O)_3](NO_3)$ {[**1**](NO_3)}, which was confirmed by ^1H NMR spectroscopy (Fig. S10). ^1H NMR (400 MHz, in D_2O): δ 1.62 (s, 15H, H of η^5-C_5Me_5).

$[Ru_2^{IV}(\eta^5\text{-}C_5Me_5)_2(\mu\text{-}O)_2](NO_3)_2$ {[2](NO_3)$_2$}. O_2 was bubbled through a H_2O solution (30 mL) of [**1**](NO_3) (381 μmol) at 0–5 °C for 30 min to form [**2**]$(NO_3)_2$. The pH of the resulting solution was adjusted to 7.2 by addition of one equivalent of NaOH (0.10 M NaOH aqueous solution, 1.88 mL, 188 μmol) to **2** because this complex is stable at neutral pH. Then the solvent was removed under reduced pressure and a brown powder was obtained (126 mg, 188 μmol, yield: 99%). ESI-MS (in H_2O): *m*/*z* 521.9 {[**2** + OH]$^+$, relative intensity (*I*) = 100% in the range of *m*/*z* 200–2000}. Anal. Calcd for $C_{20}H_{30}N_2O_8Ru_2\cdot NaOH\cdot H_2O$: C, 34.99; H, 4.84; N, 4.08. Found: C, 34.98; H, 4.50; N,

4.43. SQUID: diamagnetic.

Isotope Labelling Experiment with $^{16}O_2$ in $H_2^{18}O$. $^{16}O_2$ was bubbled through a $H_2^{18}O$ solution (500 μL) of [**1**](NO_3) (2.4 μmol) at 0–5 °C to form $[Ru_2^{IV}(\eta^5\text{-}C_5Me_5)_2(\mu\text{-}^{18}O)_2](NO_3)_2$ {[^{18}O-labeled **2**]$(NO_3)_2$}. ESI-MS (in $H_2^{18}O$): *m*/*z* 527.9 ([^{18}O-labeled **2** + $^{18}OH]^+$, I = 100% in the range of *m*/*z* 200–2000). This result indicates that oxido ligands of **2** can be exchanged for external $H_2^{18}O$.

Isotope Labelling Experiment with $^{18}O_2$ in $H_2^{16}O$. $^{18}O_2$ was bubbled through a H_2O solution (500 μL) of [**1**](NO_3) (2.4 μmol) at 0–5 °C to form $[Ru_2^{IV}(\eta^5\text{-}C_5Me_5)_2(\mu\text{-}O)_2](NO_3)_2$ {[**2**]$(NO_3)_2$}. ESI-MS (in H_2O): *m*/*z* 521.9 ([**2** + OH]$^+$, I = 100% in the range of *m*/*z* 200–2000). This result indicates that oxido ligands of ^{18}O-labeled **2** can be exchanged for external H_2O.

Catalytic Oxidation of CH_4 by O_2 with Complex 2 in H_2O under UV Light Irradiation. A H_2O solution (2.0 mL) of [**2**]$(NO_3)_2$ (1.0 mM) under CH_4 (4 MPa) and O_2 (2 MPa) atmospheres was irradiated by UV light (250–385 nm, 15 mW/cm^2) for 5 h. After the reaction, 1.0 mL of the resulting solution was passed through a silica gel column (300 mg) to remove the Ru complex(es). The metal-free aqueous solution was adjusted to 2.0 mL by addition of H_2O, then analysed by GC-MS. The amounts of CH_3OH and HCHO were determined by calibration curves. The turnover numbers (TONs) of CH_3OH and HCHO were estimated as follows: (mol of CH_3OH)/(mol of **2**) for CH_3OH and (mol of HCHO)×2/(mol of **2**) for HCHO. The TONs of CH_3OH and HCHO were determined as 1.1 and 3.0, respectively. Thus, the total TON was calculated as 4.1. No HCOOH was observed by GC-MS whose limit of detection was less than 0.02 mM, corresponding to a TON of 0.06. The yields of CH_3OH and HCHO based on CH_4 were determined to be 0.12 and 0.17%, respectively. After more than 5 h irradiation of UV light, complex **2** was decomposed, which was confirmed by ESI-MS (Fig. S8). Control experiments were conducted without **2**, UV light (250–385 nm), CH_4, or O_2, showing no product formation. When visible light (385–740 nm, 15 mW/cm^2) was used instead of UV light, no reaction occurred.

Isotope Labelling Experiment for Catalytic Oxidation of CH_4 by O_2 with Complex 2 in $H_2^{18}O$ under UV Light Irradiation. A $H_2^{18}O$ solution (300 μL) of [**2**]$(NO_3)_2$ (1.0 mM) under CH_4 (4 MPa) and $^{16}O_2$ (2 MPa) atmospheres was irradiated by UV light (250–385 nm) for 5 h. After the reaction, the resulting solution was passed

through a silica gel column (60 mg) to remove the Ru complex(es), and the metal-free aqueous solution was analysed by GC-MS, showing $CH_3{}^{16}OH$ formation without $CH_3{}^{18}OH$.

Catalytic Oxidation of CH_3OH by O_2 with Complex 2 in H_2O under UV Light Irradiation. A H_2O solution (2.0 mL) of [**2**]$(NO_3)_2$ (1.0 mM) and CH_3OH (2.0 mM) was irradiated by UV light (250–385 nm, 15 mW/cm^2) for 5 h. After the reaction, 1.0 mL of the resulting solution was passed through a silica gel column (300 mg) to remove the Ru complex(es). The metal-free aqueous solution was adjusted to 2.0 mL by addition of H_2O, then analysed by GC-MS. The amounts of CH_3OH and HCHO were determined by calibration curves. The TON of HCHO was determined as 1.1 and it was confirmed that the unreacted CH_3OH remained as is. No HCOOH was observed by GC-MS whose limit of detection was less than 0.02 mM, corresponding to a TON of 0.04. Control experiments were conducted without **2**, UV light (250–385 nm), CH_3OH, or O_2, showing no product formation. When visible light (385–740 nm, 15 mW/cm^2) was used instead of UV light, no reaction occurred.

Catalytic Oxidation of C_2H_6 by O_2 with Complex 2 in H_2O under UV Light Irradiation. A H_2O solution (2.0 mL) of [**2**]$(NO_3)_2$ (1.0 mM) under C_2H_6 (2 MPa) and O_2 (1 MPa) atmospheres was irradiated by UV light (250–385 nm, 15 mW/cm^2) for 5 h. After the reaction, 1.0 mL of the resulting solution was passed through a silica gel column (300 mg) to remove the Ru complex(es). The metal-free aqueous solution was adjusted to 2.0 mL by addition of H_2O, then analysed by GC-MS. The amounts of C_2H_5OH and CH_3CHO were determined by calibration curves. The TONs of C_2H_5OH and CH_3CHO were estimated as follows: (mol of C_2H_5OH)/(mol of **2**) for C_2H_5OH and (mol of CH_3CHO)×2/(mol of **2**) for CH_3CHO. The TONs of C_2H_5OH and CH_3CHO were determined as 0.31 and 0.46, respectively. Thus, the total TON was calculated as 0.77. A trace amount of CH_3COOH was observed. Control experiments were conducted without **2**, UV light (250–385 nm), CH_4, or O_2, showing no product formation. When visible light (385–740 nm, 15 mW/cm^2) was used instead of UV light, no reaction occurred. Because the maximum pressure of C_2H_6 is 2 MPa in technical aspects, C_2H_6 oxidation was not conducted at the same pressure as CH_4 oxidation (4 MPa).

Computational Method. All full-optimized structures were obtained by using the UB3LYP functional[2,3] as implemented in the Gaussian 16 packages.[4] The SDD basis set[5]

was used for the Ru atom, and the D95** basis set[6] was used for the C, O, and H atoms. By calculating the analytical harmonic vibration frequencies, we confirmed that the obtained local minima and transition states have none and one imaginary frequency, respectively. The contribution of zero-point energy corrections and the Grimme-D3 dispersion energy corrections[7] were included to predict reliable energetics. To obtain UV spectra and excited states, TD-DFT[8] calculations were carried out.

Table S1 Calculated Mulliken spin densities for the H atom abstraction of CH_4 in the excited triplet state ($\mathbf{T_{27}}$) and the ground singlet state ($\mathbf{S_0}$).

excited triplet ($\mathbf{T_{27}}$)	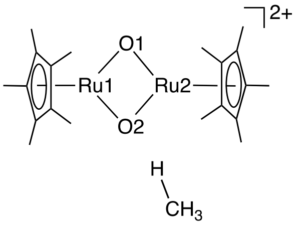	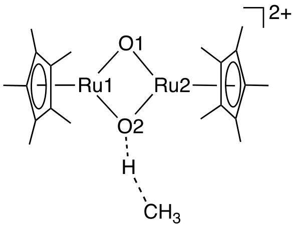	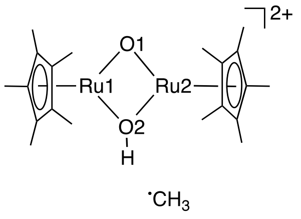
	**^{3}R***	**^{3}TS***	**^{3}P***
Ru1	0.63	0.70	0.91
Ru2	0.56	0.13	–0.07
O1	0.23	0.35	0.23
O2	0.24	0.32	0.06
C	0.00	0.52	1.00
ground singlet ($\mathbf{S_0}$)	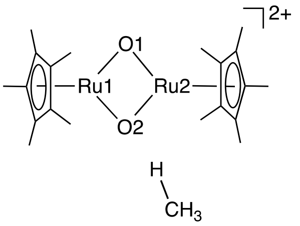	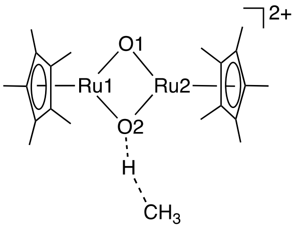	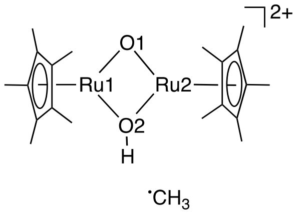
	**^{1}R**	**^{1}TS**	**^{1}P**
Ru1	–0.79	–0.57	–0.68
Ru2	0.77	–0.07	–0.07
O1	0.01	–0.28	–0.30
O2	0.03	0.25	0.03
C	0.00	0.61	0.94

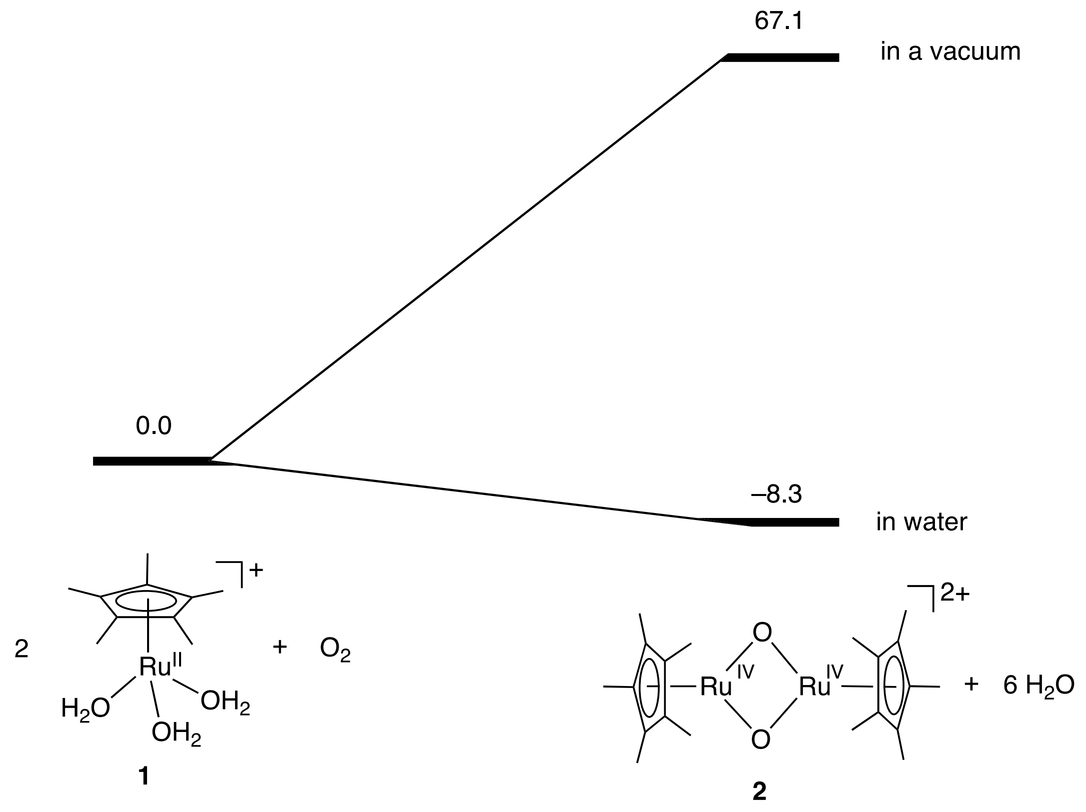

Fig. S1 Energy profiles for the formation of the bis(μ-oxido)Ru_2^{IV} complex **2** starting from the Ru^{II} triaqua complex **1** in the ground states in vacuum and water using the polarizable continuous model (PCM). The relative energies calculated from the dissociation limit of the bimolecular **1** and O_2 are given in units of kcal/mol.

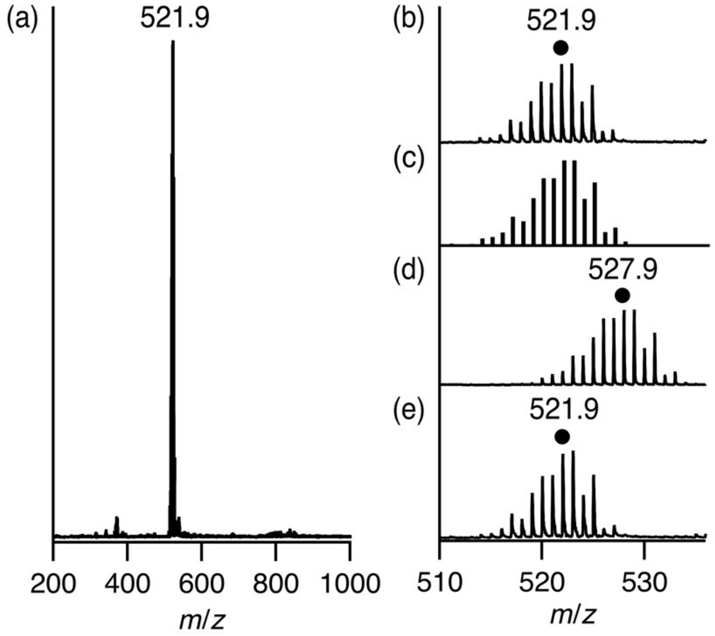

Fig. S2 (a) A positive-ion ESI mass spectrum of **2** in H_2O. (b) A signal at *m/z* 521.9 for [**2** + OH]$^+$. (c) A calculated isotopic distribution for [**2** + OH]$^+$. (d) A positive-ion ESI mass spectrum of ^{18}O-labeled **2** in $H_2{}^{18}O$, which is obtained from the reaction of **1** with O_2 in $H_2{}^{18}O$. (e) A positive-ion ESI mass spectrum of **2** in H_2O, which is obtained from the reaction of **1** with $^{18}O_2$ in H_2O.

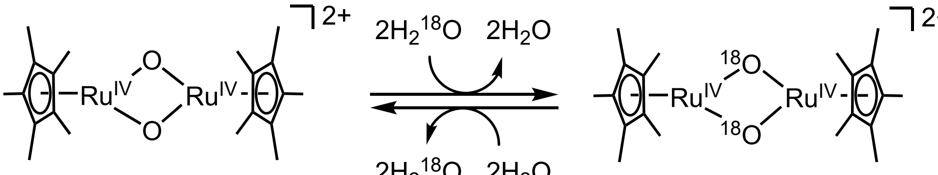

2 ^{18}O-labelled **2**

Fig. S3 Exchange of $H_2{}^{18}O$ with oxido ligands in a bis(μ-oxido) $Ru^{IV}{}_2$ complex, and *vice versa*.

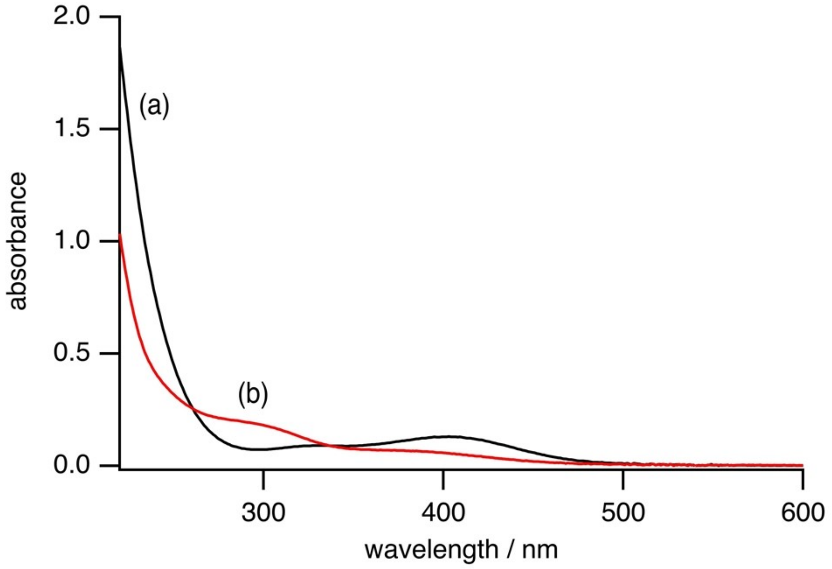

Fig. S4 UV-vis spectra of (a) $[Ru^{II}(\eta^5\text{-}C_5Me_5)(H_2O)_3]^+$ (**1**) in H_2O (1.0 mM) (black line) under a N_2 atmosphere and (b) $[Ru_2^{IV}(\eta^5\text{-}C_5Me_5)_2(\mu\text{-}O)_2]^{2+}$ (**2**) in H_2O (0.50 mM) (red line) under an O_2 atmosphere.

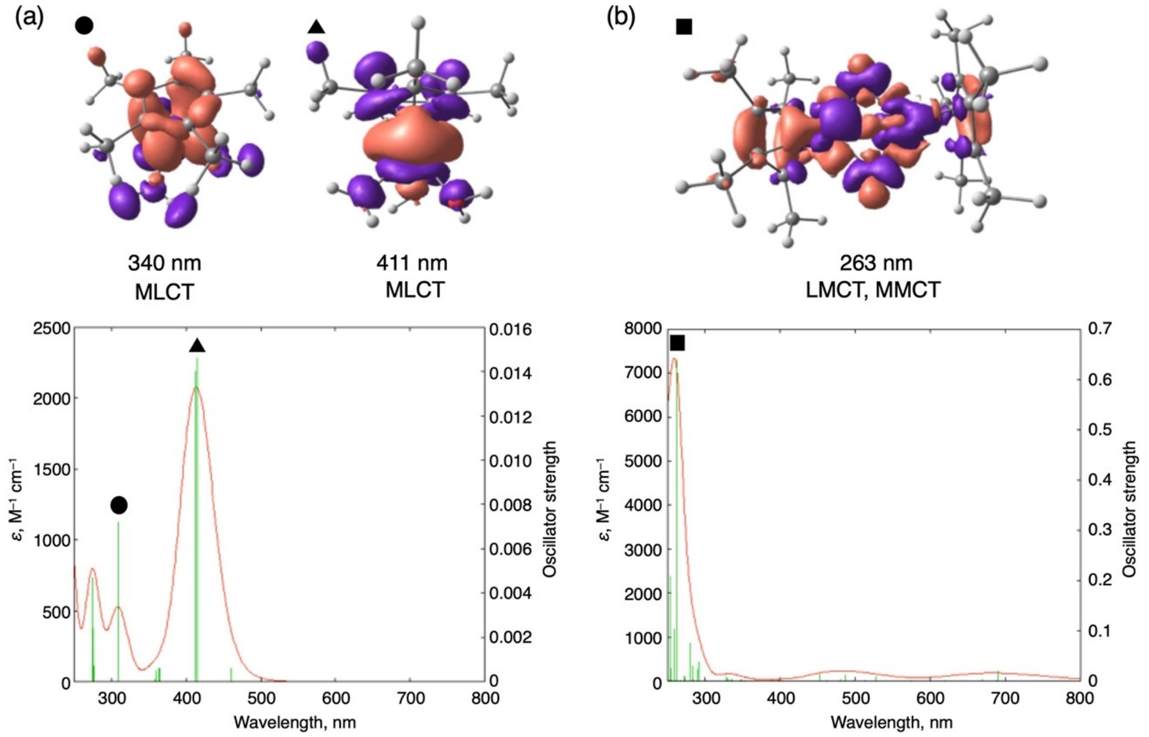

Fig. S5 Computed UV-vis spectra of (a) complex **1** and (b) complex **2** with electron-density difference pictures. Orange: electron-density decrease; purple: electron-density increase. As a result of the charge transfer, the electron density in the orange region is decreased while that in the purple region is increased. Absorptions shown in the calculated spectra as the circle, triangle, and rectangle were assigned to respective transitions depicted as orbital distributions above. MLCT: metal-to-ligand charge-transfer. LMCT: ligand-to-metal charge-transfer. MMCT: metal-to-metal charge transfer.

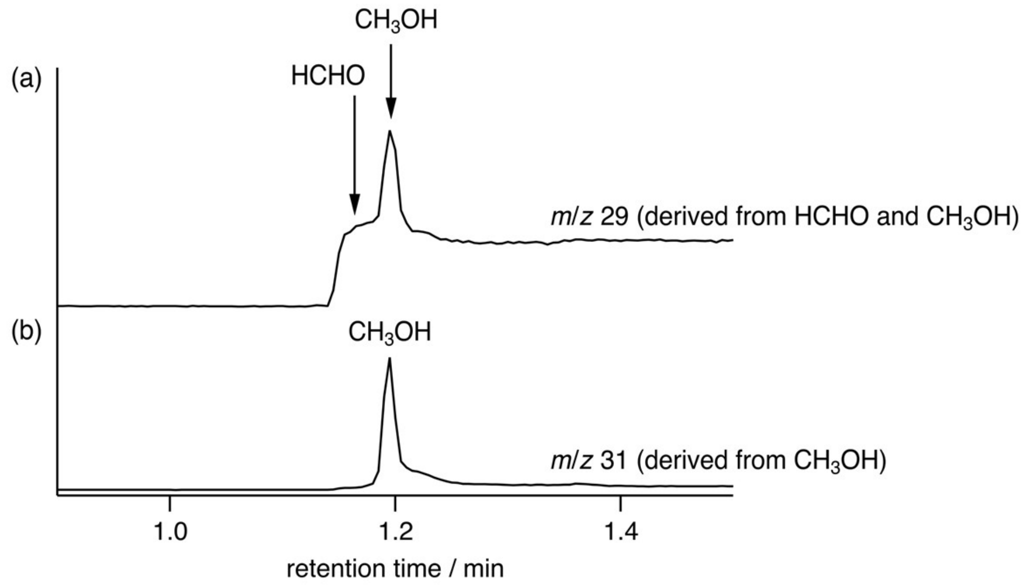

Fig. S6 GC-MS data of CH_3OH and HCHO obtained from the photo-induced oxidation of CH_4 by $[Ru_2^{IV}(\eta^5\text{-}C_5Me_5)_2(\mu\text{-}O)_2]^{2+}$ (**2**) in H_2O. GC-MS analysis for (a) *m*/*z* 29 derived from HCHO and CH_3OH and (b) *m*/*z* 31 derived from CH_3OH.

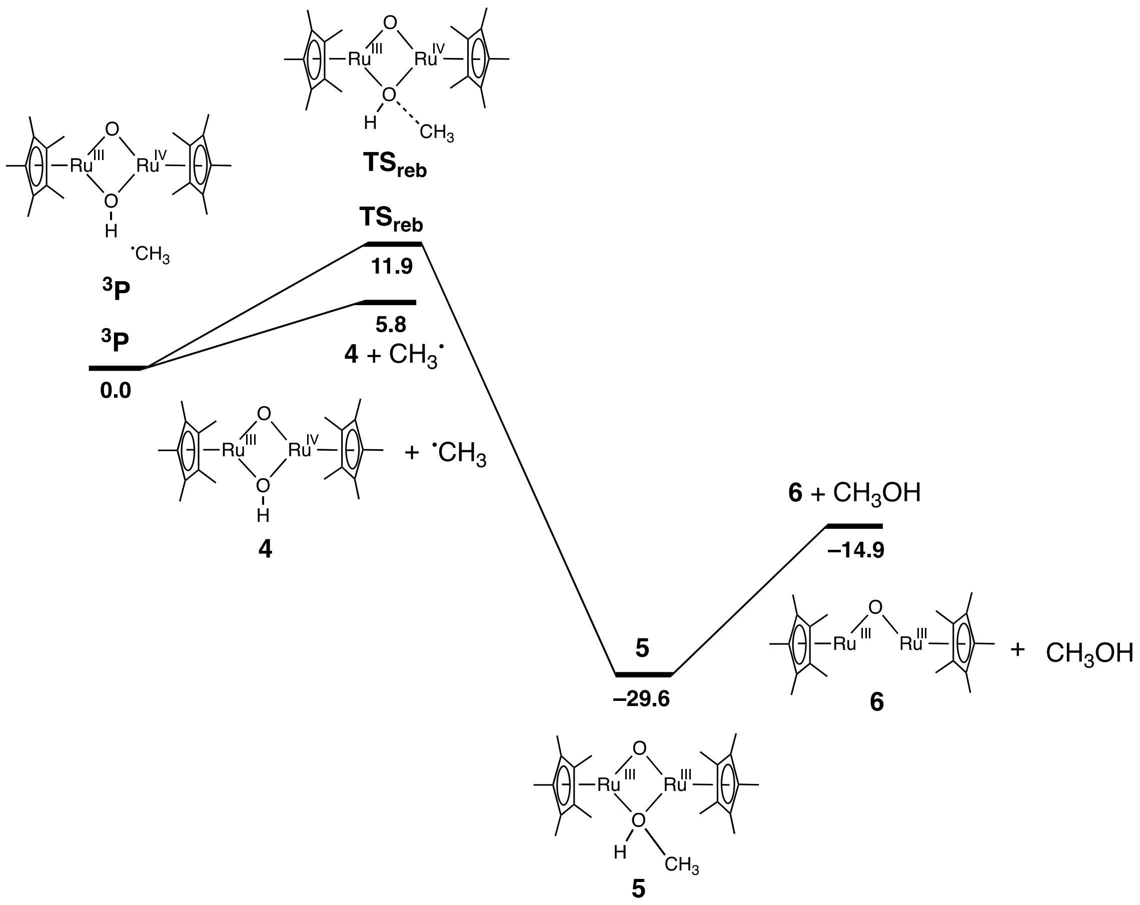

Fig. S7 Energy profile for the reaction of the (μ-hydroxido)(μ-oxido)$Ru_2^{III,IV}$ complex **4** and methyl radical ($CH_3^•$) in the triplet state. The relative energies calculated from the complex ^{3}**P** are given in units of kcal/mol.

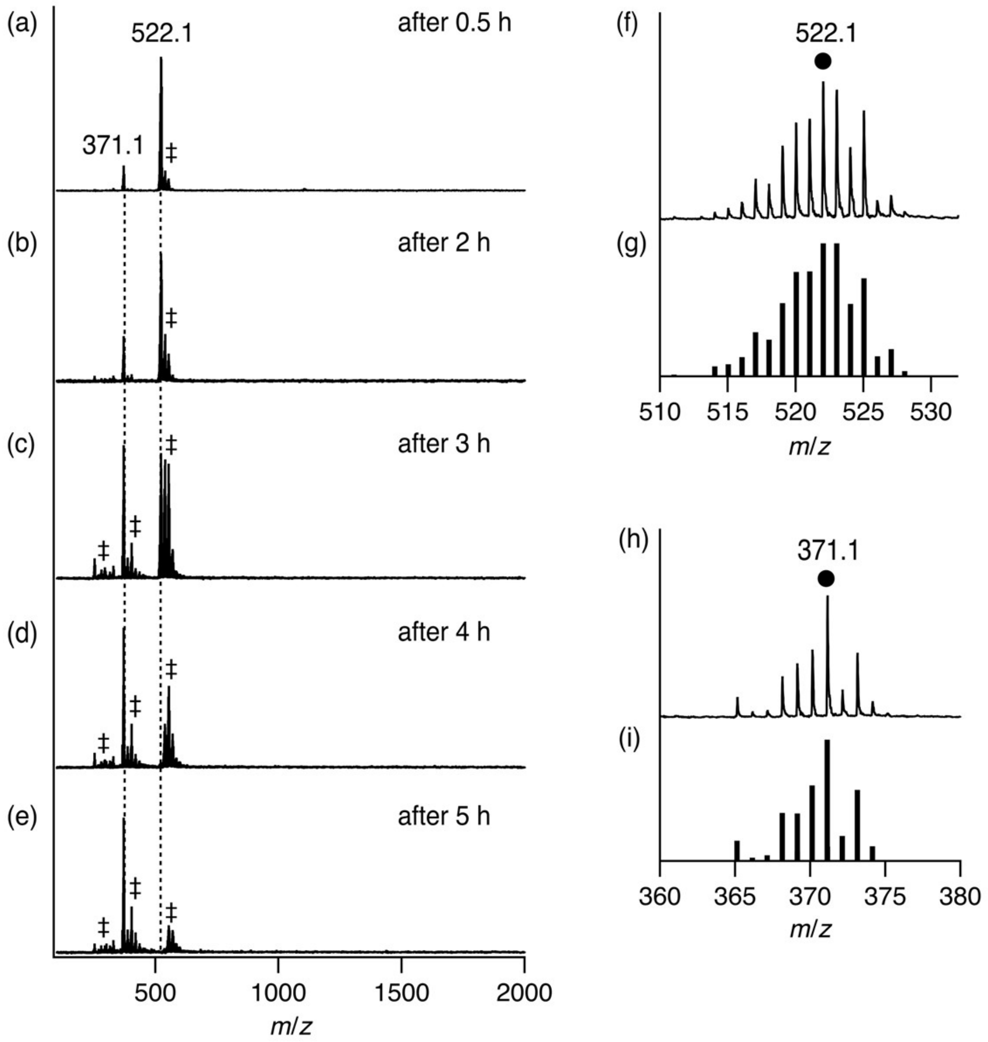

Fig. S8 Positive-ion ESI mass spectra obtained from the reaction of $[Ru_2^{IV}(\eta^5\text{-}C_5Me_5)_2(\mu\text{-}O)_2]^{2+}$ (**2**) with CH_4 and O_2 in H_2O under light irradiation for (a) 0.5 h, (b) 2 h, (c) 3 h, (d) 4 h and (e) 5 h. (f) A signal at *m*/*z* 522.1 for [**2** + OH]$^+$. (g) A calculated isotopic distribution for [**2** + OH]$^+$. (h) A signal at *m*/*z* 371.1 for $[Ru^{II}$(tetramethylfulvene)$(\eta^5\text{-}C_5Me_5)]^+$. (i) A calculated isotopic distribution for $[Ru^{II}$(tetramethylfulvene)$(\eta^5\text{-}C_5Me_5)]^+$. ‡: Unidentified species.

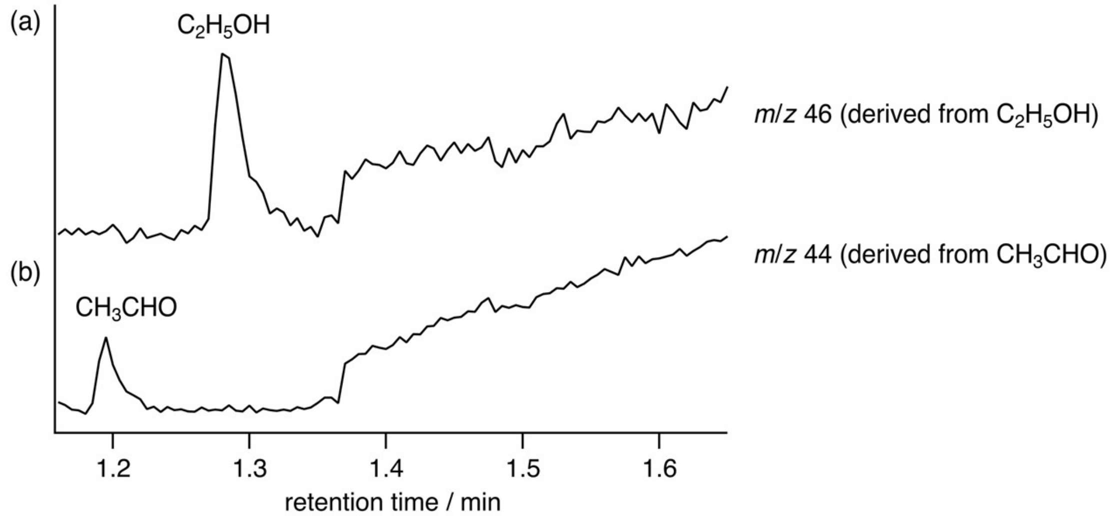

Fig. S9 GC-MS data of C_2H_5OH and CH_3CHO obtained from the photo-induced oxidation of C_2H_6 by $[Ru_2^{IV}(\eta^5\text{-}C_5Me_5)_2(\mu\text{-}O)_2]^{2+}$ (**2**) in H_2O. GC-MS analysis for (a) *m*/*z* 46 derived from C_2H_5OH and (b) *m*/*z* 44 derived from CH_3CHO.

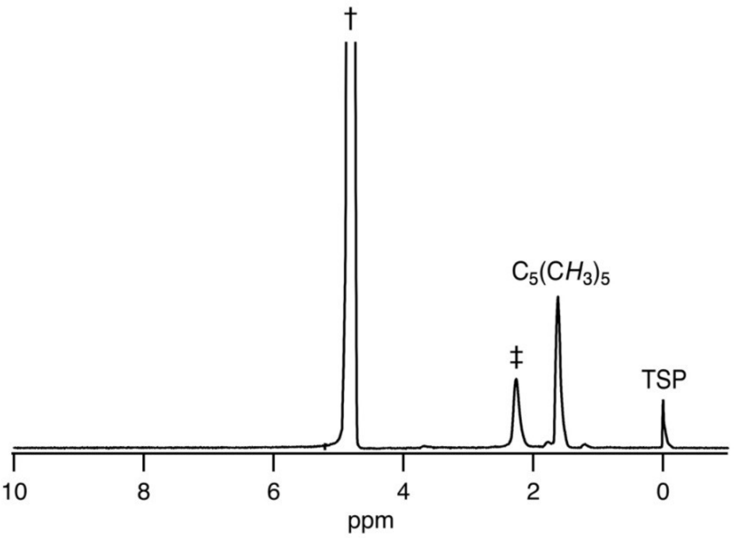

Fig. S10 ^{1}H NMR spectrum of $[Ru^{II}(\eta^5\text{-}C_5Me_5)(H_2O)_3](NO_3)$ {[**1**](NO_3)} in D_2O under a N_2 atmosphere, in which H_2O ligands should be D_2O ligands because of the use of D_2O as a solvent. 3-(Trimethylsilyl)-propionic-2,2,3,3-d_4 acid sodium salt (TSP), referenced with the methyl proton resonance set at 0.00 ppm. †: water. ‡: free acetonitrile released from Ru center, which was confirmed by comparison with free acetonitrile.

References

1 M. Takenaka, M. Kikkawa, T. Matsumoto, T. Yatabe, T. Ando, K.-S. Yoon and S. Ogo, *Chem. Asian J.*, 2018, **13**, 3180–3184.

2 A. D. Becke, *J. Chem. Phys.*, 1993, **98**, 5648–5652.

3 C. Lee, W. Yang and R. G. Parr, *Phys. Rev. B*, 1988, **37**, 785–789.

4 Gaussian 16, Revision C.01, M. J. Frisch, G. W. Trucks, H. B. Schlegel, G. E. Scuseria, M. A. Robb, J. R. Cheeseman, G. Scalmani, V. Barone, G. A. Petersson, H. Nakatsuji, X. Li, M. Caricato, A. V. Marenich, J. Bloino, B. G. Janesko, R. Gomperts, B. Mennucci, H. P. Hratchian, J. V. Ortiz, A. F. Izmaylov, J. L. Sonnenberg, D. Williams-Young, F. Ding, F. Lipparini, F. Egidi, J. Goings, B. Peng, A. Petrone, T. Henderson, D. Ranasinghe, V. G. Zakrzewski, J. Gao, N. Rega, G. Zheng, W. Liang, M. Hada, M. Ehara, K. Toyota, R. Fukuda, J. Hasegawa, M. Ishida, T. Nakajima, Y. Honda, O. Kitao, H. Nakai, T. Vreven, K. Throssell, J. A. Montgomery, Jr., J. E. Peralta, F. Ogliaro, M. J. Bearpark, J. J. Heyd, E. N. Brothers, K. N. Kudin, V. N. Staroverov, T. A. Keith, R. Kobayashi, J. Normand, K. Raghavachari, A. P. Rendell, J. C. Burant, S. S. Iyengar, J. Tomasi, M. Cossi, J. M. Millam, M. Klene, C. Adamo, R. Cammi, J. W. Ochterski, R. L. Martin, K. Morokuma, O. Farkas, J. B. Foresman, and D. J. Fox, Gaussian, Inc., Wallingford CT, 2016.

5 D. Andrae, U. Häußermann, M. Dolg, H. Stoll and H. Preuß, *Theor. Chim. Acta*, 1990, **77**, 123–141.

6 T. H. Dunning, Jr. and P. J. Hay, *in Modern Theoretical Chemistry,* Ed. Schaefer, H. F. III, Vol. 3 (Plenum, New York, 1977), pp. 1–28.

7 S. Grimme, J. Antony, S. Ehrlich and H. Krieg, *J. Chem. Phys*., 2010, **132**, 154104.

8 E. Runge and E. K. U. Gross, *Phys. Rev. Lett.*, 1984, **52**, 997–1000.
